# Influence of defects on structural colours generated by laser-induced ripples

**DOI:** 10.1038/s41598-019-56638-x

**Published:** 2020-01-09

**Authors:** Stella Maragkaki, Christian A. Skaradzinski, Ralf Nett, Evgeny L. Gurevich

**Affiliations:** 1 Chair of Applied Laser Technologies, Ruhr-Universität Bochum, Universitätsstraße 150, 44801 Bochum, Germany; 2 Institute of Electronic Structure and Laser, Foundation for Research and Technology (IESL-FORTH), 71110 Heraklion, Crete Greece

**Keywords:** Materials science, Optics and photonics

## Abstract

The colourisation of metallic surface which appears due to periodic surface patterns induced by ultrashort laser pulses is studied. Ripples due to the sub-micrometer size of their period act as a diffraction grating, generating structural colours. Carefully chosen strategy of the laser spot scanning allows us to mimic the nanostructures responsible for structural colours of some flowers on the metal substrate. We investigate the correlation between the colourising effects and the artificially-induced defects in the ripples structure and show that these defects can make the colours observable in a larger range of viewing angles. Further we address the influence of the processing parameters on the spectral profile of the reflected light.

## Introduction

**Figure 1:**
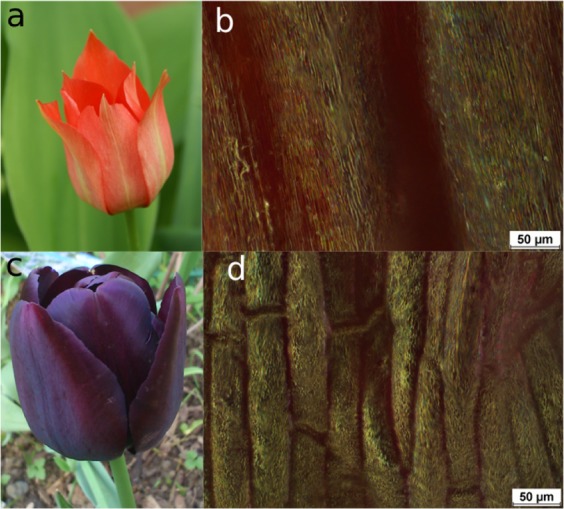
Microscopic structures of some flowers. *Tulipa linifolia*: (**a**) flower; (**b**) microscopic dark field image; Tulip *Queen of the Night*: (**c**) flower; (**d**) microscopic dark field image. The microscopic images are done with *Nikon Eclipse LV100* optical microscope in the manual z-scan mode, the length scale is 50 *μ*m.

Nature provides numerous examples of vivid and vibrant structural colours originated from complex interactions between light and sophisticated nanostructures generated in the natural world^1–4^. These natural colours are the inspiration of biomimetic research on micro/nano fabrication with a significant potential for sustainable production and recycling^5^. Such colour phenomena are usually based on the diffraction of incident light by periodic structures, which characteristic period is of the same order of magnitude as the wavelength of the scattered light. The first artificial structural colours were made by Gabriel Lippmann by photoreduction of silver salt in the photographic emulsion in the nodes of interference patterns. This method was the first example of colour photography, which was distinguished by the Nobel Prize in 1908^6^. However, the photographs produced in this way could be observed only from the same viewing angle, from which the Lippmann plates were exposed, otherwise the colours changed. Structural colours in nature are iridescent, i.e., are changed with the viewing angle, which is important e.g., for flowers sending visual signals to bees^3^. However the viewing angle, at which the iridescent colours can be observed, must be large enough to attract more bees. This angle-independency can be achieved by a certain degree of disorder in the natural photonic structures^3^. This can be achieved either by irregularities^2^ or by superimposing periodic structures on submicrometer and on 10–100 *μ*m scales, see Fig. 1 (see also Fig. 1 in ref. ^3^ for more images).

For the last decades, ultrashort laser pulses have been proven to be the best candidate for precise micro/nano machining altering the optical properties of the laser-processed surface such as reflectance and colour. Moreover, laser processing offers high precision, clean process without pigments and chemicals and no contact with the sample is necessary^7,8^. The drawback of the laser direct writing of photonic structures is the low productivity of such methods. However self-organised surface structures like laser-induced periodic structures (LIPSS) or ripples offer the possibility of a one-step rapid production of periodic structures^9–12^.

It is very important though to be able to control the size of the period as the spectral regions of the observed colours are directly depending on the spatial periods of the ripples^13^. Experiments show that an easy method to control the ripples period under suitable laser parameters such as the laser fluence and the scanning speed is the laser wavelength^13,14^. In terms of applications, the colourisation of metallic surfaces is promising in anti-counterfeiting labelling, optical encryption, laser marking and optical data storage^15–17^. However, from the practical point of view the laser-induced ripples are useless for colour marking because they act as a diffraction grating: each incident wavelength is mostly reflected at certain angles, so that the observed colour is changing with the viewing angle.

The viewing angle is a very important factor and can be defined as the maximum range of angles, at which the colours of a surface can be viewed. Recent experimental results regarding to the relationship between the colours and the viewing angle give the evidence of the grating diffraction effect originating the angle-dependence colourizing^18,19^, but the role of disorder in the LIPSS for the colour formation remains unclear. Such a quantitatively experimental study would give insights on the critical factors that affect the colourizing phenomena and therefore, would provide guidelines for improving the controllability of these phenomena caused by surface periodic structures. The aim of this work is to find correlation between the observed colours and LIPSS topography and to understand, how to control the spectral characteristics of the colours. Commonly, large areas of regular diffraction nanogratings are reported in the literature as the most suitable surface profile for the colourizing effect^20–24^. Obviously, the full coverage with regular LIPSS has drawbacks like e.g. long processing time and additional restriction of the laser parameters required for the pattern homogeneity^20^. In this paper we will show that a nearly ideal periodic LIPSS pattern is not optimal for structural colours and defects on the surface can facilitate the observation of vivid colours at a larger range of viewing angles. The influence of disorder on the structural colours is usually discussed in the context of biology^3,4^. Here we use a combination of self-organized LIPSS and controllable direct laser writing to mimic floral nanostructures with some degree of disorder, as that, responsible for structural colours^3^, and show that areas not fully covered with LIPSS reveal special colour effects with much broader diffraction spectrum.

## Results

### Fabrication of microstructures for structural colouration

One of the ways how to produce structural colours and mimic the coloured surfaces observed in nature is to cover the surface with micro/nano gratings. In our experiments we use LIPSS induced on a stainless steel surface by femtosecond laser pulses with a periodicity slightly smaller than the laser wavelength. More information regarding to the material composition is given in the Supplementary Material. Diode-pumped Yb:KYW thin-disc femtosecond laser (*JenLas D2*.*fs*, *Jenoptik*) operating at $1025\,nm$ wavelength and 30 kHz repetition rate is used in the experiments. The 300-femtosecond laser pulses are linearly polarized and the direction of the polarization is adjusted by a $\lambda /2$ wave plate. The beam is directed and focused onto the sample by a galvanometric system (*SCANLab*) equipped with an f-theta lens with focal length at $63\,mm$. The beam radius ${\omega }_{0}$ was calculated to be 14.7 *μ*m. The peak fluence ${\Phi }_{P}$ is calculated by using the relation: ${\Phi }_{P}=2{E}_{P}/\pi {\omega }_{0}^{2}$, where *E*_*P*_ is the laser pulse energy. After the processing, the surface topology was studied by scanning electron microscopy (SEM, *Zeiss EVO MA 15*) and atomic force microscopy (AFM, *Nanoscope 5*, *Bruker Corp*.).

In contrast to other publications, we do not aim to produce a homogeneous ripple structure but study how defects and gaps in the LIPSS influence the colouration. The study is motivated by the following arguments: (1) rapid surface coverage with gaps between laser tracks increases the production rate and allows up-scaling of the process without increase in the laser repetition rate and average power. Moreover, if the quality of LIPSS can be sacrificed, the processing parameters can be optimized to increase the productivity. (2) The structural colours in nature are much more vivid than that obtained from structures created in the laboratory. But the natural structures are formed by self-organisation^4^ and are full of defects comparing to nearly ideal laboratory specimens. (3) The structural colours in flowers are formed by periodic nanostructures on a sub-micrometer length scale superimposed by gaps or other quasi-periodic structures with a larger period^3^. So we assume that the defects play an important role in the colouration process.

Toward this approach, we realized several square areas on a $600\,m{m}^{2}$ polished surface of stainless steel (each $25\,m{m}^{2}$) covered with ripples. The one surface is fully covered with certain vertical overlap (named as *A*), while other surfaces are only partly covered with ripples (named as *B*, *C*, and *D*), or completely covered but without vertical overlap between the horizontal laser scans (named as *E*). This is realized by altering the distance between the scanning lines, so that the percentage of ripples on each area can be controlled. In the areas *A*, *B*, *D*, and *E* the orientation of the ripples is parallel to the scanning line direction, in the area *C* it is perpendicular. The summary of the areas is given in the Table 1.

**Table 1:** Summary of the laser parameters for the five different areas covered with LIPSS, with Φ_*P*_ the peak fluence, width the distance between the scanning lines, line spacing the width of the non-irradiated line between the scanning lines, N/spot the number of pulses per spot in the horizontal and vertical direction, ripples orient.

Area	Φ_*P*_ (*J*/*cm*^2^)	width (*μ*m)	line spacing (*μ*m)	horiz. (N/spot)	vertical (N/spot)	ripples orient.	patterned area (%)
A	0.41	4	—	6	4	$\parallel$	100
B	0.65	30	6	14	—	$\parallel$	87
C	0.65	30	6	14	—	$\perp$	87
D	0.65	40	14	14	—	$\parallel$	65
E	0.65	24	—	14	—	$\parallel$	100

The orientation of ripples in respect to the scanning line and patterned area the percentage of area covered with ripples.

### Analysis of the structural colours

**Figure 2:**
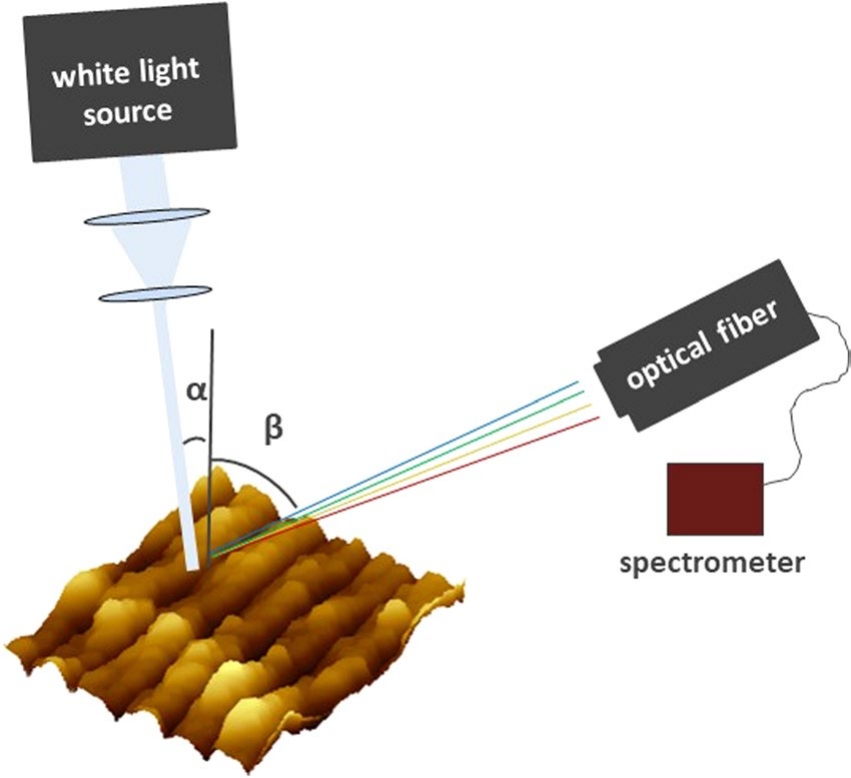
Schematic representation of the setup for the spectroscopic analysis of the diffracted light upon illumination of LIPSS surface with a white light source. The spot of the illumination source covers the whole patterned area.

The optical properties of the LIPSS covering the samples are analysed in a set-up shown in Fig. 2, which enables observation of the structural colours revealed after illuminating the surface with white light. In our experiments the white light source *SCHOTT KL 1500 electronic* was used for the illumination. The colours were recorded (1) by spectrometer (*Ocean Optics*, *USB2000*+) and (2) photographically. In the latter case the fiber of the spectrometer was substituted by a white screen, and the light pattern was captured with a photo camera (*Panasonic*, *DMC-G70KA Lumix G*). The same settings of the camera (exposure time, aperture, colour balance) were used for each capture. The spot of the illumination source was chosen to be approximately $20\,m{m}^{2}$ in order to cover the whole patterned area. At the same time the surrounding surface of the sample was covered with a black paper in order to guarantee that the spectra are collected only from the patterned area.

After the laser processing, LIPSS or ripples appear on the surface. One cannot treat these structures as an ideal periodic grating due to structural defects and partial coverage. Hence the real LIPSS can be decomposed into three types of surface structures with different optical properties:

1. irregular periodic pattern, which can be described as a superposition of a set of ideal LIPSS with continuously changing period, acting as an ideal diffraction grating;
2. highly-reflecting not-processed area providing specular reflection of the incident light;
3. point-like defects in the periodic structure scattering light in all directions proportional to $I(\theta )\propto (1+{co}{{s}}^{2}\,\theta )$, where *θ* is the angle between the incident and the scattered light.

We suppose that the optical properties of the LIPSS surface can be seen as a superposition of light scattered by these three patterns different types of the surface. In the following discussion, the importance of these factors for the colouration effect will be evaluated.

As it is schematically represented in Fig. 2, when a periodically nanostructured surface is illuminated by a white-light source at an incident angle *α*, the central wavelength of the diffracted light *λ* in a certain angle *β* is given by the diffraction equation: $m\lambda =\Lambda (\sin \,\alpha +\,\sin \,\beta )$ with ripples spacing $\Lambda$ and diffraction order *m*.

**Figure 3:**
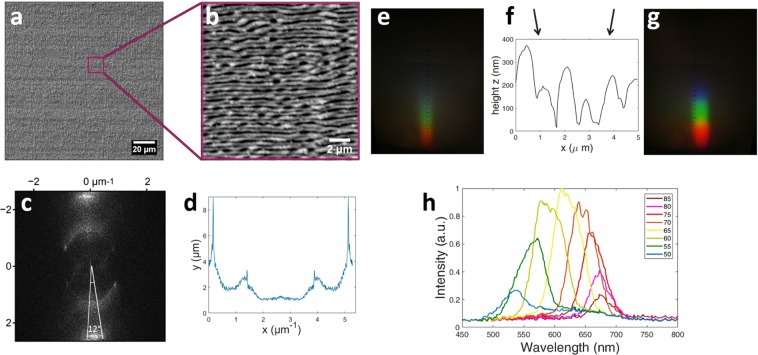
Area A (see Table 1) 100% covered with ripples induced by $N=6$ femtosecond laser pulses per spot in the horizontal direction and $N=4$ in the vertical direction. The LIPSS period is approx. 800 nm. (**a**) SEM micrograph of area A, (**b**) higher magnification of the box on *a* (**c**) the 2D-FFT of the image a without the labels, (**d**) the 2D-FFT profile of image *c* (**e**,**g**) are captures of the diffracted light after illuminating the whole area A with a white light source, (**f**) the AFM profile of the LIPSS appear on area A. The arrows in (**f**) indicate the direction of the white light source. Capture (**e**) is the result of illumination from the left side (left arrow), while capture (**g**) is the result of illumination from the right side (right arrow). In both cases the surface is illuminated in a direction perpendicular to the LIPSS orientation. Plot (**h**) shows the spectrum analysis of diffracted light from area A by using the configuration shown in Fig. 2. Each plot corresponds to the spectrum observed under a different diffraction angle *β*. The legend on the right side of the plot shows the angle *β.*

**Figure 4:**
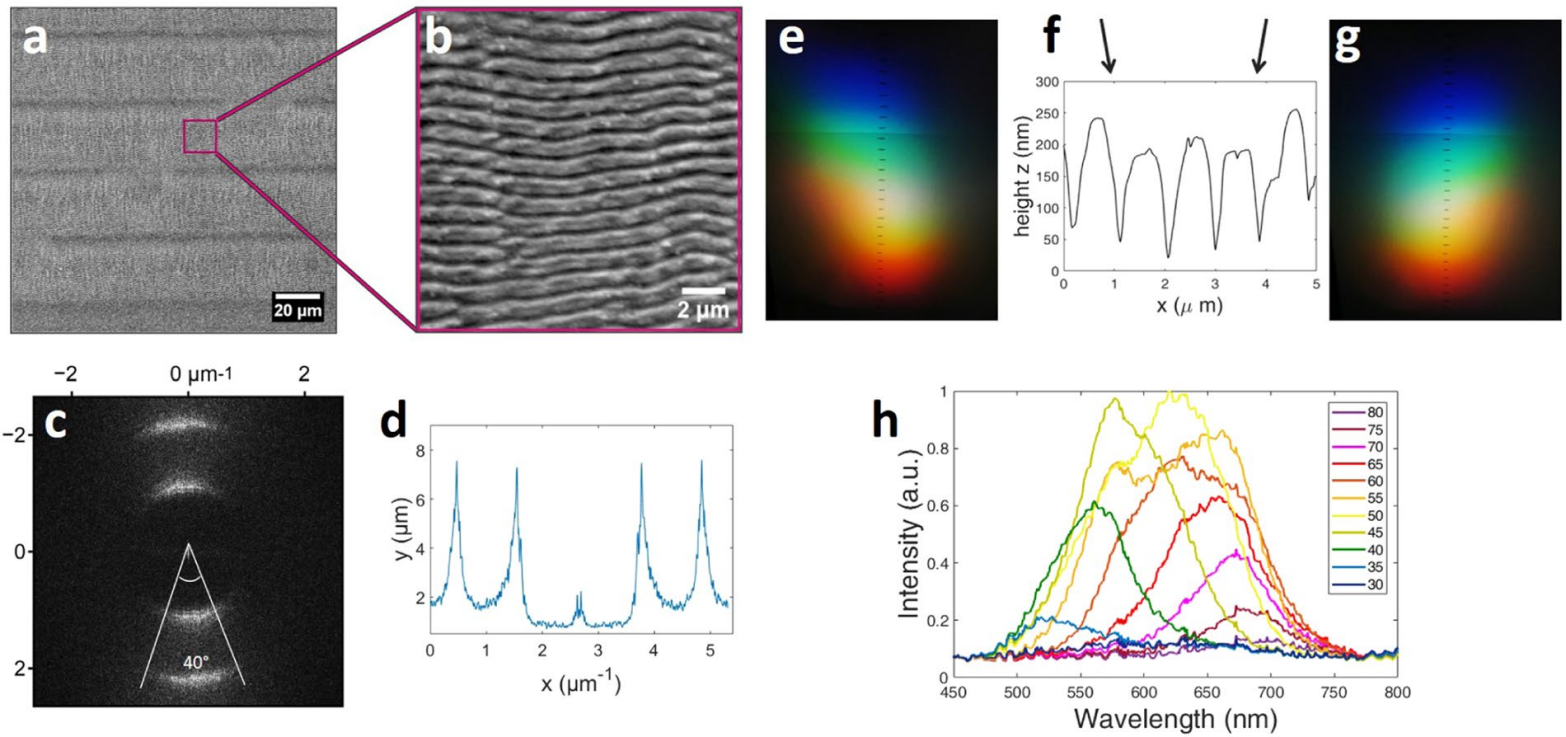
Area B (see Table 1) 87% covered with ripples induced by $N=14$ femtosecond laser pulses per spot in the horizontal direction. There is not vertical overlapping between the scanning line. The LIPSS period is approx. 900 nm. (**a**) SEM micrograph of area B, (**b**) higher magnification of the box on a (**c**) the 2D-FFT of the image a without the labels, (**d**) the 2D-FFT profile of image c (**e**,**g**) are captures of the diffracted light after illuminating the whole area B with a white light source, (f) the AFM profile of the LIPSS appear on area B. The arrows in (**f**) indicate the direction of the white light source. Capture (**e**) is the result of illumination from the left side (left arrow), while capture (**g**) is the result of illumination from the right side (right arrow). In both cases the surface is illuminated in a direction perpendicular to the LIPSS orientation. Plot (**h**) shows the spectrum analysis of diffracted light from area B. Each plot corresponds to the spectrum observed under a different diffraction angle *β*. The legend on the right side of the plot shows the angle *β.*

Firstly, the spectrometer in the setup (Fig. 2) is replaced by a white paper, on top of which the diffracted colours appear and captured with a camera. The illumination direction was always chosen to be perpendicular to the ripple orientation. The results are shown in Figs. 3e,g and 4e,g. The reason we present two different images for each case will be explained later in this section. The results reveal a broader spectrum of diffracted colours over a wider area when the surface is not fully covered with ripples (Fig. 4). This was an unexpected result, because it shows that a broader spectrum can be revealed when only a part of the surface is covered with nanostructures. The next step was to further characterize the structures so as to investigate the origins of this effect. The surface morphology of the obtained structures is analysed by means of scanning electron (SEM) and atomic force (AFM) microscopy and the optical properties by optical spectroscopy in order to quantify the colour effects. The periodicity of the patterns is analysed by means of two-dimensional fast Fourier transformation (2D-FFT).

### Characterization

SEM analysis (Figs. 3a,b and 4a,b) shows the topography of the regular periodic structures formed in each area. The SEM micrographs (Figs. 3a and 4a) are transferred in the frequency domain through 2D-FFT analysis, which are shown in Figs. 3c and 4c. The FFT profiles along the lines corresponding to the bisectors of the angles marked in Figs. 3c and 4c are shown in Figs. 3d and 4d respectively. The structure of the FFT profiles consists of three main parts:

1. *Zero frequency*. The central part corresponding to zero frequency of the pattern. This part can be either elongated as in Fig. 4c due to long-period waving of the ripples, which can be clearly seen in Fig. 4b, or have additional maxima at approximately 120 and 40 degrees as in Fig. 3c, which appear due to bifurcations of the ripples and point-like defects. The formation of these point-like defects on the neighbouring ripples influences each other, hence quadratic and hexagonal structures appear locally on the surface (see Fig. 3b), as it also happens in hydrodynamics^25^, which play important role in the laser-induced melt flows^24,26^.
2. *First period*. The first period corresponding to the period of the LIPSS. The angular width of this part of the spectrum was referred as *dispersion in the LIPSS orientation angle (DLOA)* in^27^ and is in the focus of this paper.
3. *Second period*. The second period corresponding to the double structure of the ripples in the SEM images, which characteristic length scale is approximately 400–450 nm.

The period of the structures consisting of arrangements of point-like defects on the neighboring ripples (denoted as part of the *zero frequency*) is close to the period of the LIPSS (*first period*). Such arrangements, when they exist, cover only a small area of the metal surface (see Fig. 3b) but the electron emission from them is strong due to their sharp topology, hence they contribution to the *first period* is overestimated and must be excluded by evaluation of the DLOA. It is interesting to note that the DLOA measurement based on the width of the *second period* is free from this influence and can also be used for the estimation of the evaluation of the LIPSS orientation dispersion.

The ripple period, which is measured through the analysis of the first period of the 2D-FFT plots is $\Lambda =800\,nm$ for the area A and $\Lambda =900\,nm$ for the area B. The zero frequency in an FFT profile represents the homogeneous intensity background and the parts of the SEM image at which there is no certain periodicity. In Fig. 3d it is almost zero while in Fig. 4d it is quite high. This verifies that in the first case the whole surface is covered with periodic structures while in the second case there are areas without a certain periodicity. This zero frequency signal is collected from the unexposed areas between the scanning lines.

**Figure 5:**
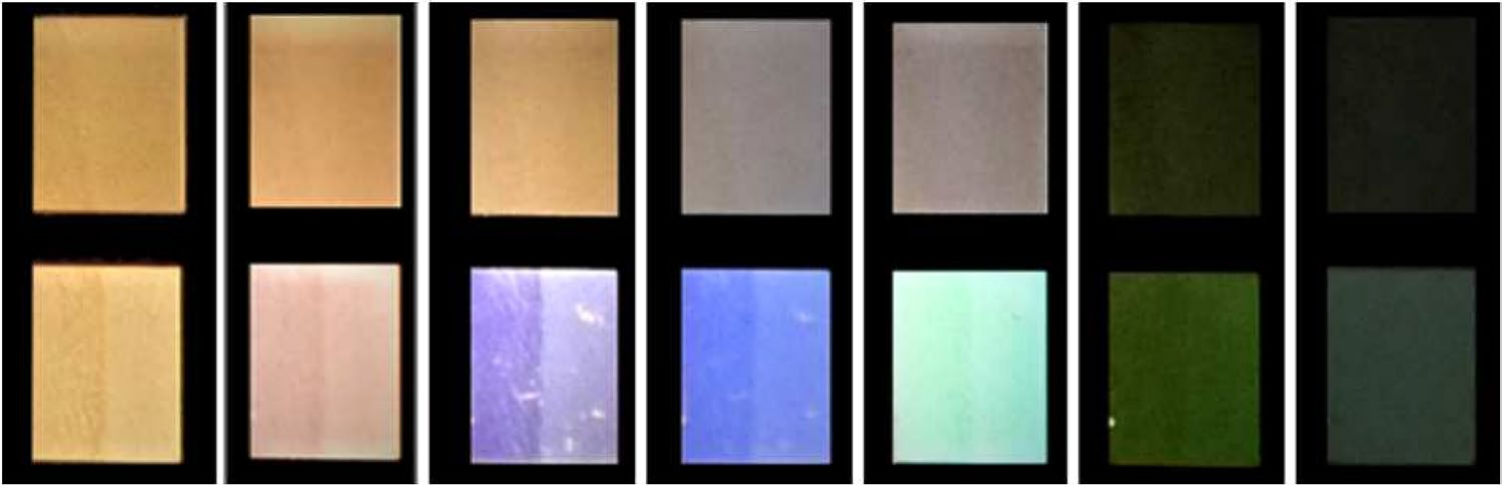
Areas A (above) and B (below) illuminated by white light and observed at different angles (which are the same for each pair of the images). The illumination parameters are the same, for the features of the LIPSS patterns see Table 1.

As mentioned we observed more vivid colour diffracted from the area, which is only partly covered with ripples (Fig. 4e,g). The rainbow-like colours in the photographs (Fig. 4e,g) appear more mixed which is the result of overlapping between two different visible spectra. Within optical spectroscopy (see setup in Fig. 2) we were able to quantify the range and intensity of colours observed in each viewing angle and verify whether the colours in area *B* are indeed more vivid in comparison to area *A*. The results are shown in Figs. 3h and 4h. Each plot corresponds to the spectrum observed under a different diffraction angle. The legend on the right part of the plot shows the angle *β* (which is pointed in Fig. 2). The incident angle was $\alpha =10^\circ$ in all cases. We can clearly observe that in area A the width of each sub-plot is much more narrow than in area B. These results show that when the surface is fully covered with ripples (area *A*) in each observation angle there is a limited range of different diffracted colours. Further, the range of different diffracted angles at which colours can be observed is limited as well. On the contrary, when only a part of the surface is covered with LIPSS (area *B*) the structural colours are observed over a wider range of viewing angles, while at each angle almost the whole spectrum in the visible regime is diffracted revealing a colourful surface. Photographic images of the fully and partly covered surfaces captured at different angles are shown in Fig. 5.

Further, we shouldn’t dismiss another advantage of the partly covered with ripples surfaces. In Fig. 3f, the AFM profile shows the cross section of the periodic structure. When we illuminated the surface from the left side (see left arrow in Fig. 3f) we observed that the colour phenomena are very intense (Fig. 3g), while in case of illuminating the surface from the opposite side (see right arrow in Fig. 3f) the colour phenomena are too weak to be observed (Fig. 3e). This can be explained by the ripples morphology (Fig. 3f). When the illumination is coming from the right side, part of the diffracted light hits the walls of the ripples which are higher in thickness and therefore the diffraction phenomenon is not equally observed from both sides. On the contrary, on the partly covered with ripples surface, where the periodic structures are more smooth and the heights are equally distributed, we observed the colour phenomena with equal intensity in both directions (see e.g. area *B* in Fig. 4e–g). A reason for this behaviour is the higher pulse overlapping in area *A*, which could result in more irregular height between the crest and valleys of the ripples.

Nevertheless, it is crucial to identify the reason the structural colours are more vivid in case of a lower percentage of ripples on the surface. Therefore, we studied the influence of the orientation and percentage of ripples on the structural colours. To study the influence of the ripples orientation on the structural colours, we realized one more squared area partly covered with ripples with the same parameters as in area B. The only difference is that here ripples are oriented perpendicular to the scanning line direction (named as area *C*). In addition, in order to study how the percentage of LIPSS on the surface can influence the observed diffracted colours we realized two more squared areas (named as *D* and *E*). Each square is covered with a different percentage of nanostructures, all the other parameters such as fluence, polarization and horizontal overlapping are kept constant. The laser parameters are shown in Table 1. Surface topology and structural colouration analysis for those areas (C, D and E) are illustrated in the Supplementary Material. The result of these additional studies show that all surfaces reveal much more vivid colours in comparison to area *A* - fully covered with ripples. Still, the spectra collected from areas *B* (87% of the surface covered with ripples), *D* (65%) and *E* ((100%) are almost the same. Therefore, the percentage of ripples doesn’t seem to play a key role. It should be clear that although both areas *A* and *E* are fully covered with ripples (see Table 1), in area *E* there is no vertical overlapping, while in area *A* the vertical overlapping is approximately 20% (4 pulses per spot), which changes completely the quality and morphology of LIPSS.

For quantitative characteristics of the LIPSS quality we measure the angle range of the 2D-FFT similar to the method suggested in the literature^20,27^. The 2D-FFT analysis plays a key role here toward understanding the mechanism responsible for the more vivid colours as will be discussed in the next section.

## Discussion

**Figure 6:**
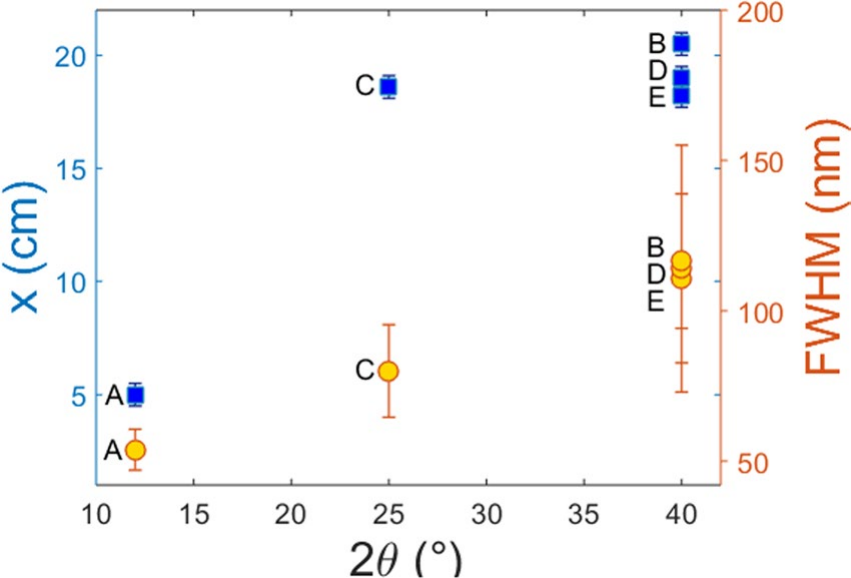
Relation between the FFT angle, the width of the coloured captures and the FWHM of the spectra. The capital letters A–E, correspond to LIPSS areas formed with different laser parameters, which are summarized in Table 1. Squares correspond to the left y axis, while dots to the right y axis.

**Figure 7:**
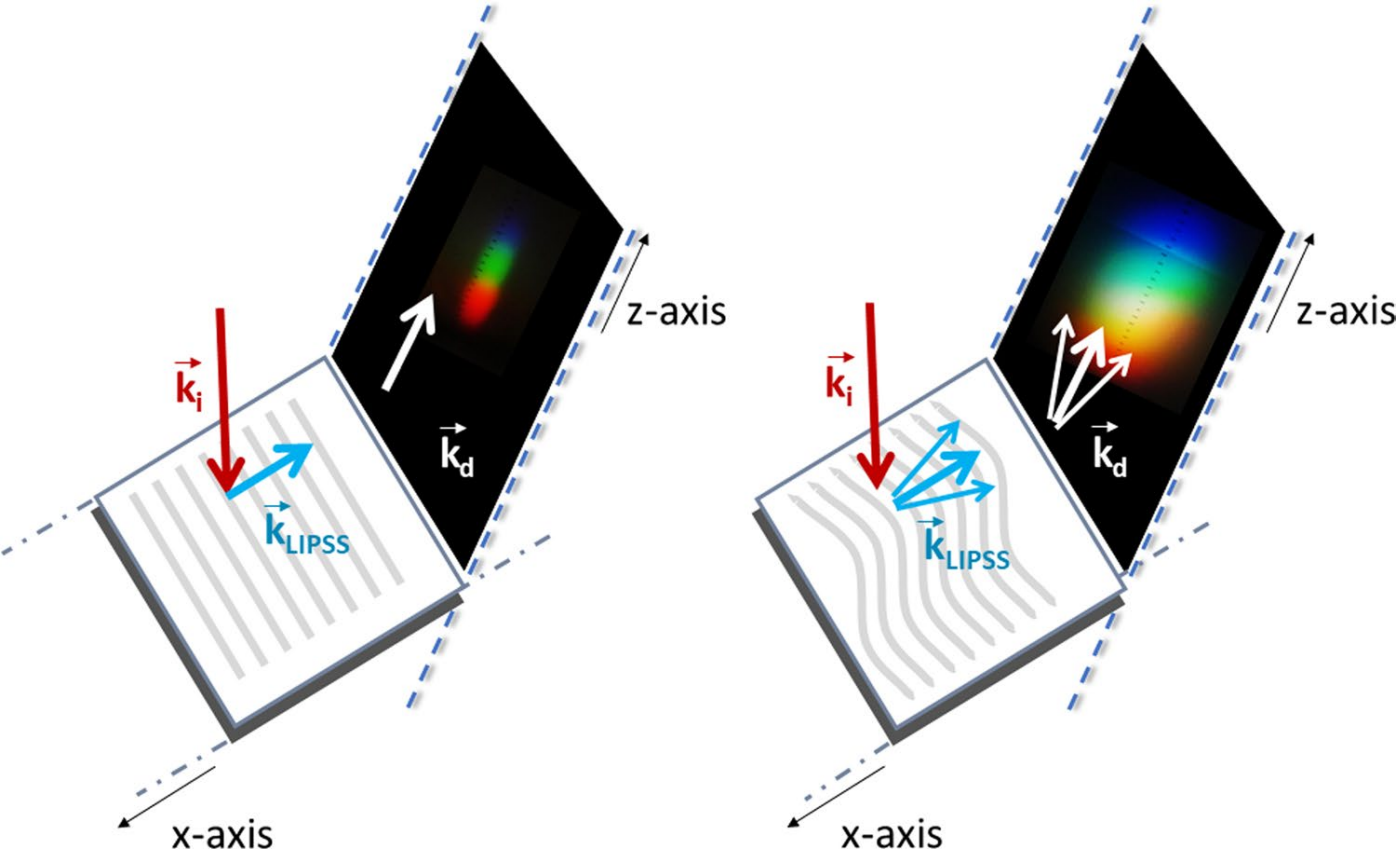
Schematic representation of the k-vectors of the diffraction phenomenon due to the presence of disorders in LIPSS orientation. The left scheme represents a symmetric periodic structures orientation. In the right scheme it is shown how the presence of disorders generates different k vectors with an effect on the final colouring spectrum width.

The idea of the colourising phenomena obtained by periodic structures is that a surface covered with LIPSS acts as a grating, where light is deflected due to interference of light reflected from each point. A characteristic of the grating orientation is the vector $\overrightarrow{k}$. In case of a grating, $\overrightarrow{k}$ is perpendicular to the direction of the fringes. A perfect grating, where all the fringes are absolutely parallel to each other should consist of one k-vector. Therefore, the number of different $\overrightarrow{k}$ on a periodic structure is a measure of the defects and different orientations exist in the periodic pattern. The two-dimensional fast-Fourier transform (2D-FFT) is a useful mathematical tool for the characterization of the grating periods, which gives information of how broad is the range of different k-vectors in the pattern. Within 2D-FFT, the SEM image is analysed in the spatial frequency domain, where the width of the peak in the k-space is related to the range of different $\overrightarrow{k}$ appear on the periodic structures (see Figs. 3c and 4c). On the other hand, a broad range of different $\overrightarrow{k}$ on a surface results in a broader scattering of light and therefore the diffracted colours appeared on the screen (see setup in Fig. 2, where the optical fiber and the spectrometer have been substituted with a white screen) are broader in the space (see e.g. the photographs in Fig. 3e,g). The relation between this width and the number of different k-vectors is in agreement with the experimental results (Fig. 6), where the width of the coloured captures *x* is increased with the FFT angle 2*θ*. In addition, if we place a spectrometer in the path of the light diffracted (Fig. 2), broad spectra are collected at different angles. The less regular LIPSS are (and therefore larger number of different k and consequently larger FFT angle), the broader is the wavelength range of each spectrum at a specific diffraction angle. For this purpose, the full width at half maximum (FWHM) of each spectrum is measured (Figs. 3h and 4h). As illustrated in Fig. 6, the highest the FFT angle, the broadest are the spectra as expected. A schematic representation of the orientation disorder on the structural colours is illustrated in Fig. 7.

The point-like defects (e.g. nanoparticles) on the contrary do not noticeably influence the optical properties of the LIPSS-covered surface. This can be concluded comparing the SEM images with the corresponding ranges of the viewing angles. For the surface with ripples and point-like defects (see Fig. 3b) the range of the viewing angles is narrow (see Fig. 3e,g), however in the defectless surface it is broad, see also in Supplementary Material for areas *C* (Fig. S8), *D* (Fig. S10) and *E* (Fig. S11). This is easy to explain by the fact that the point defects cover only a small fraction of the LIPSS and the intensity of light scattered by a point-like scattering center decreases rapidly with the distance. This is also reflected in the FFT images in sub-figures 3d compared to 4d (see also Supplementary Material): although visually the area in the SEM image in Fig. 3b looks more disordered than in the other images, the corresponding first-order peak amplitudes in the FFT spectra are small. This small amplitude of the broad Fourier-transformed first-order peak contributes less to the scattered light intensity^9^ than the high amplitudes of the FFT peaks corresponding to the wavy LIPSS in other images.

In conclusion, it is observed that the colours subjectively seen as more vivid when the spectra are broader. On the other hand, as mentioned, the broader spectra are a results of LIPSS structures with higher order of defects and less regularity in the orientation and the period. This disorder in the orientation of the periodic structures could be responsible for the resulting colourisation^28,29^. Therefore, we assume that the defected LIPSS structures reveal more vivid colours. With respect to this approach, partly-covered with LIPSS areas are more suitable for the formation of irregularities. An argument for this behaviour is that in areas fully covered with LIPSS the overlapping rate between the scanning lines is high. Accordingly, each laser-patterned line serves as a pre-pattern for the next line leading to an increase of the regularity. To conclude, nanostructures generated by ultrashort pulses produce coloured patterns on a metallic surface by diffraction process and can be designed as the colours of a painting. The careful selection of the laser parameters as laser pulse overlapping and the laser polarization enable the formation of different colours, which can be used for colourful marking. In contrast to usual strategy covering the 100% of the surface with LIPSS, we demonstrate that partly coverage is advantageous. The advantage of this technology for systematic and industrial use relies on two important factors, the reduced production time and the reproducibility. The reason is that only a part of the surface should be covered with LIPSS, therefore the process is much faster. The reproducibility is stronger because in fully-covered surfaces the overlapping is very high in both directions; therefore the obtained structures are more sensitive to pulse energy alterations in contrast to partially-covered surfaces where there is not vertical overlapping. Additionally, colours are more vivid because the defects in the ripples structure improve the spectral profile of the reflected light and they can be observed over a broader range of viewing angles.

## Supplementary information

Supplementary Information.
