# Supplementary material for: Influence of defects on structural colours generated by laser-induced ripples
Source: Sci Rep. 2020 Jan 9;10:53. doi: 10.1038/s41598-019-56638-x (PMC6952428; doi:10.1038/s41598-019-56638-x)
Supplement: Supplementary file 1 — Supplementary Information. [file 41598_2019_56638_MOESM1_ESM.pdf]

Influence of defects on structural colours generated by laser-induced ripples

Stella Maragkaki[1,2,*], Christian A. Skaradzinski[1], Ralf Nett[1], and Evgeny L. Gurevich[1]

[1]Chair of Applied Laser Technologies, Ruhr-Universitaet Bochum, Universitaetsstrasse 150, 44801 Bochum, Germany

[2]Institute of Electronic Structure and Laser, Foundation for Research and Technology (IESL-FORTH), 71110, Heraklion, Crete, Greece

*marag@iesl.forth.gr

Supplementary Material

Material composition

The material used is steel which was mechanically polished. The thickness of the material is 1.5 mm. The structural analysis of the bulk material was analyzed through electron dispersive x-ray spectroscopy (EDX) and x-ray photoelectron spectroscopy as shown in Fig. S1 and Fig S2 respectively.

An EDX (Fig. S1) and XPS analysis (Fig. S2) show that the material used here is particularly stainless steel due to the percentage of chromium in the bulk material. The EDX analysis shows that the material consists of 74% of Fe, 14% of Cr, 9% of Ni, 2:5% of Si and 0:5% of Mo (Fig.S1). In addition, XPS spectroscopy was performed (Fig.S2) which has a very high depth resolution and reveals more in depth information of the chemical compositions on the surface.

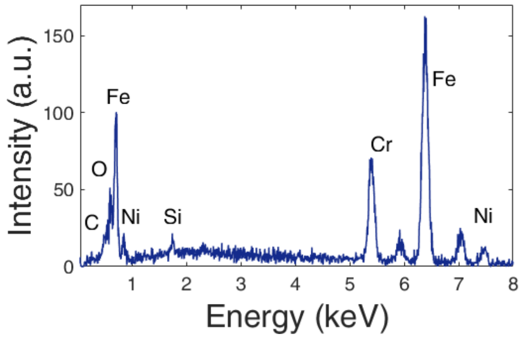

Figure S1: EDX spectra of polished stainless steel surface

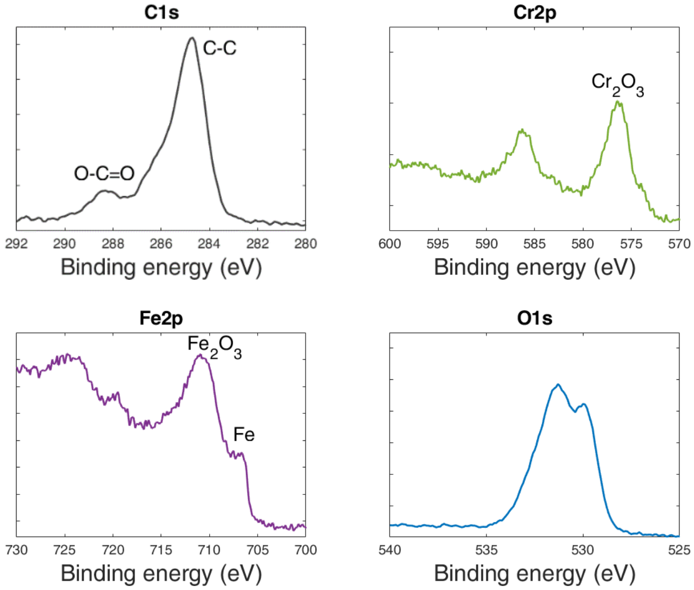

Figure S2: XPS spectra of polished stainless steel surface

Structural colors with different parameters

Five different areas have been irradiated. The repetition rate, laser wavelength and laser spot are kept constant. The peak fluence is smaller in area A because is the only surface with vertical overlapping between the scanning lines, therefore by keeping the fluence constant the dose would be much higher in comparison to the other areas leading to strong ablation.

Table S1: Summary of the laser parameters

Area	Φ_P (J/cm^2)	Width (μm)	Line spacing (μm)	Horiz. (N/spot)	Vertical (N/spot)	Ripples orientation	Patterned area (%)
**A**	0.41	4	-	6	4	∥	100
**B**	0.65	30	4	14	-	∥	87
**C**	0.65	30	4	14	-	⊥	87
**D**	0.65	40	14	14	-	∥	65
**E**	0.65	24	-	14	-	∥	100

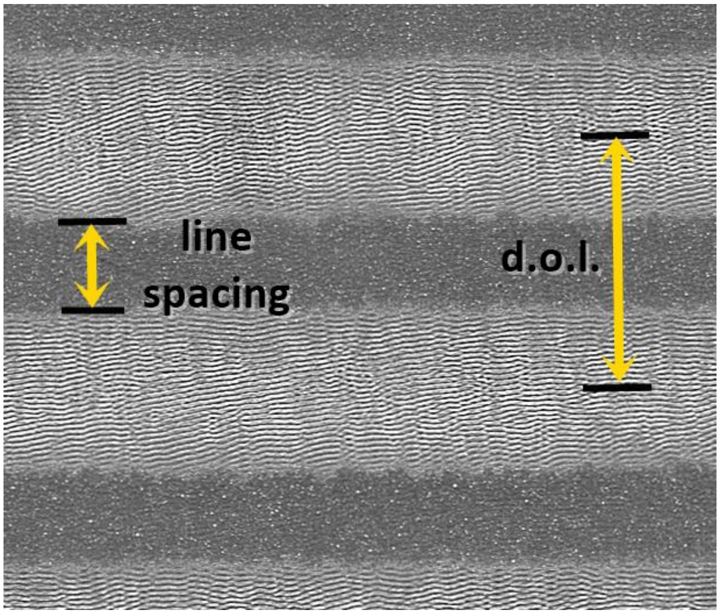

Figure S3: Schematic illustration of the scanning parameters. Line spacing represents the width of the non-irradiated line between the scanning lines and d.o.l. or width represents the distance of scanning lines from center to center

Area **A**, 100% covered with LIPSS with 20% vertical overlapping

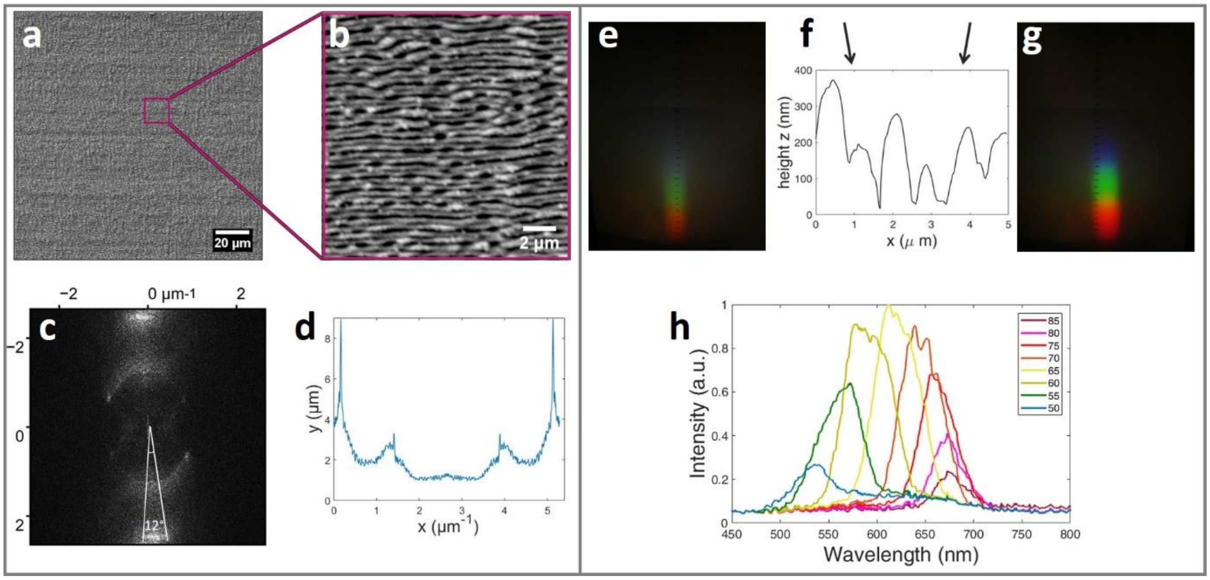

Figure S4: Area A (see table S1) 100% covered with ripples induced by N=6 femtosecond laser pulses per spot in the horizontal direction and N=4 in the vertical direction. The LIPSS period is approx. 800 nm. (a) SEM micrograph of area A, (b) higher magnification of the box on (a), (c) the 2D-FFT of the image (a) without the labels, (d) the 2D—FFT profile of image (c), (e) and (g) are captures of the diffracted light after illuminating the whole area A with a white light source, (f) the AFM profile of the LIPSS appear on area A. The arrows in (f) indicate the direction of the illumination source. Capture (e) is the result of illumination from the left side (left arrow), while capture (g) is the result of illumination from the right side (right arrow). In both cases the surface is illuminated with a direction perpendicular to the LIPSS orientation. (h) spectrum analysis of diffracted light from area A by using the configuration shown in Fig. 2 (manuscript). Each plot corresponds to the spectrum observed under a different diffraction angle β.

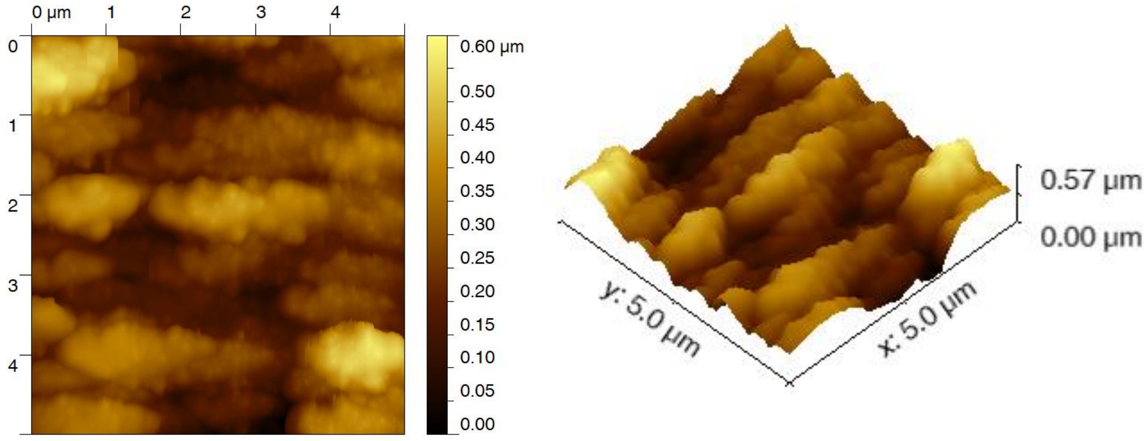

Figure S5: AFM profile of the area A (Fig. S4), 2D in (a) and 3D representation in (b). The plot of the AFM profile is presented in Fig. S4(f).

Area **B**, 87% covered with LIPSS

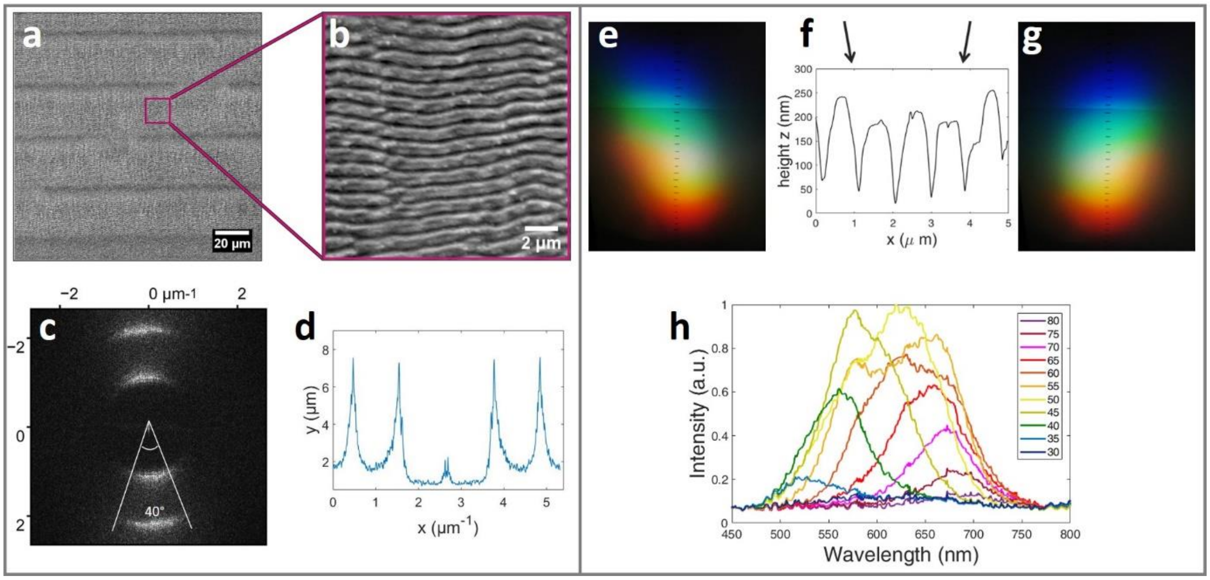

Figure S6: Area B (see table S1) 87% covered with ripples induced by N=14 femtosecond laser pulses per spot in the horizontal direction and no overlapping in the vertical direction. The LIPSS period is approx. 900 nm. Detailed description of each image is given in caption of Fig. S4.

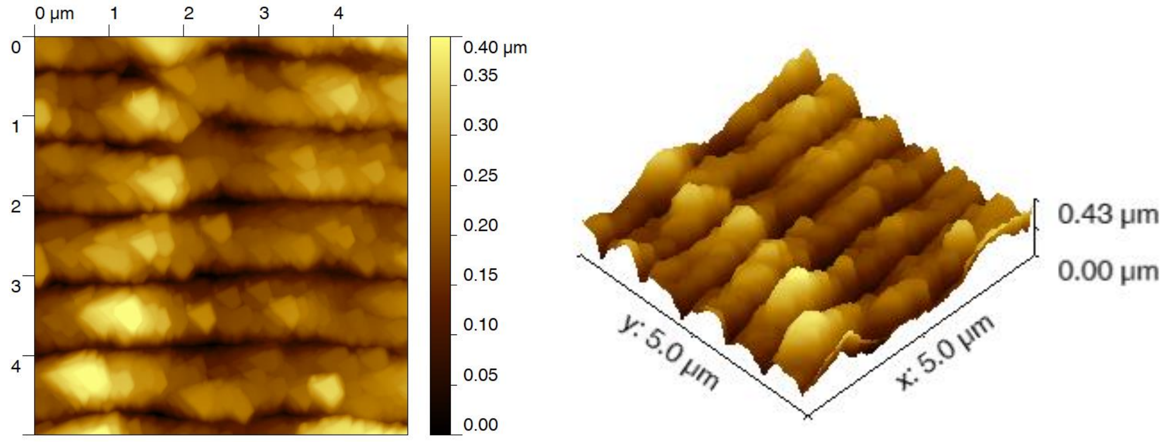

Figure S7: 2D (a) and 3D (b) AFM profile of the area B (Fig. S6). The plot of the AFM profile is presented in Fig. S6(f).

Area **C**, 87% covered with LIPSS (orientation is perpendicular in comparison to all the other areas)

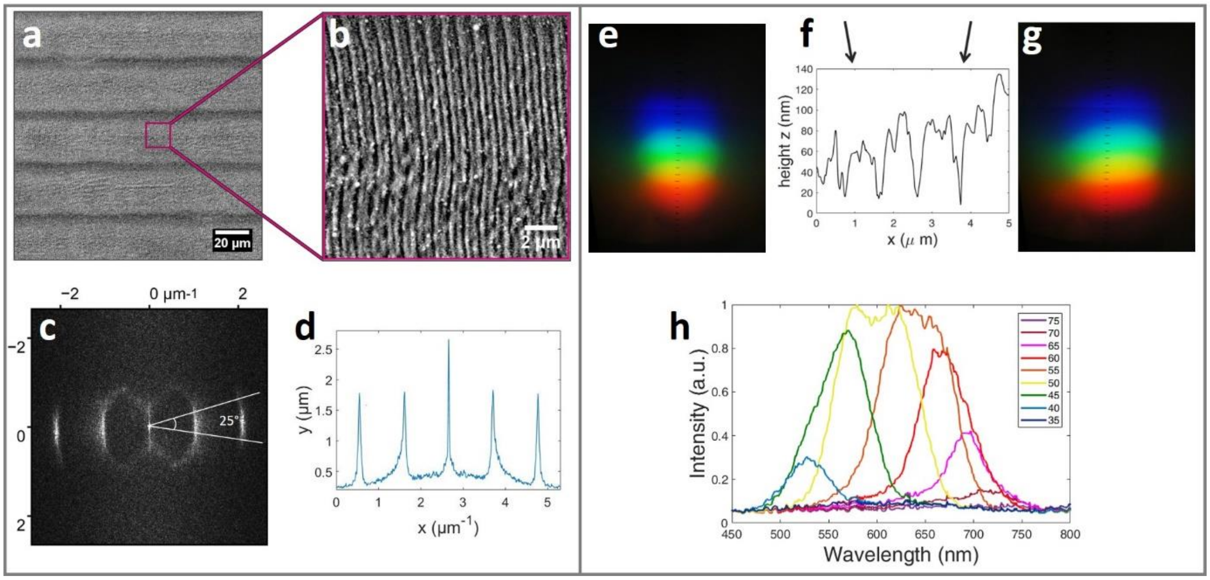

Figure S8: Area C (see table S1) 87% covered with ripples induced by N=14 femtosecond laser pulses per spot in the horizontal direction and no overlapping in the vertical direction. The LIPSS period is approx. 900 nm and the orientation perpendicular to the scanning line direction. Detailed description of each image is given in caption of Fig. S4.

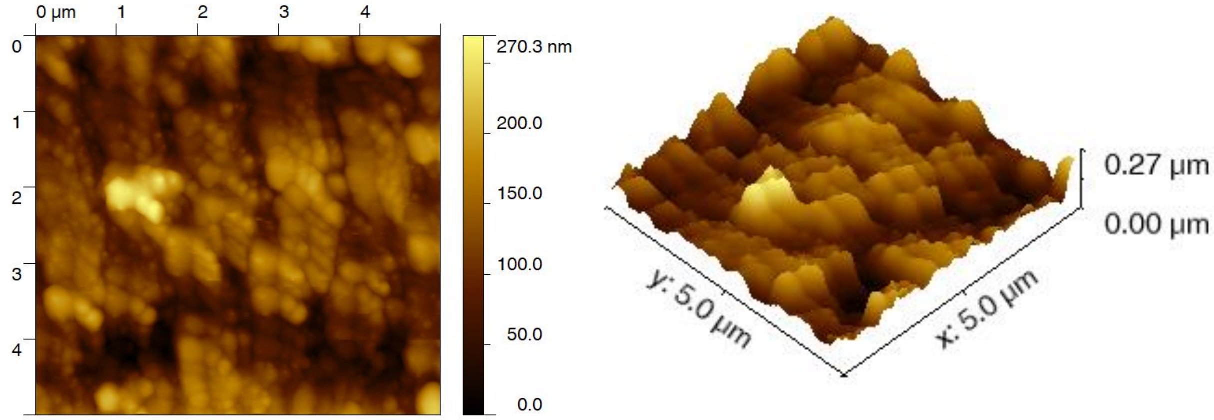

Figure S9: AFM profile of the area C (Fig. S8), 2D in (a) and 3D representation in (b). The plot of the AFM profile is presented in Fig. S8(f).

Area **D**, 65% covered with LIPSS

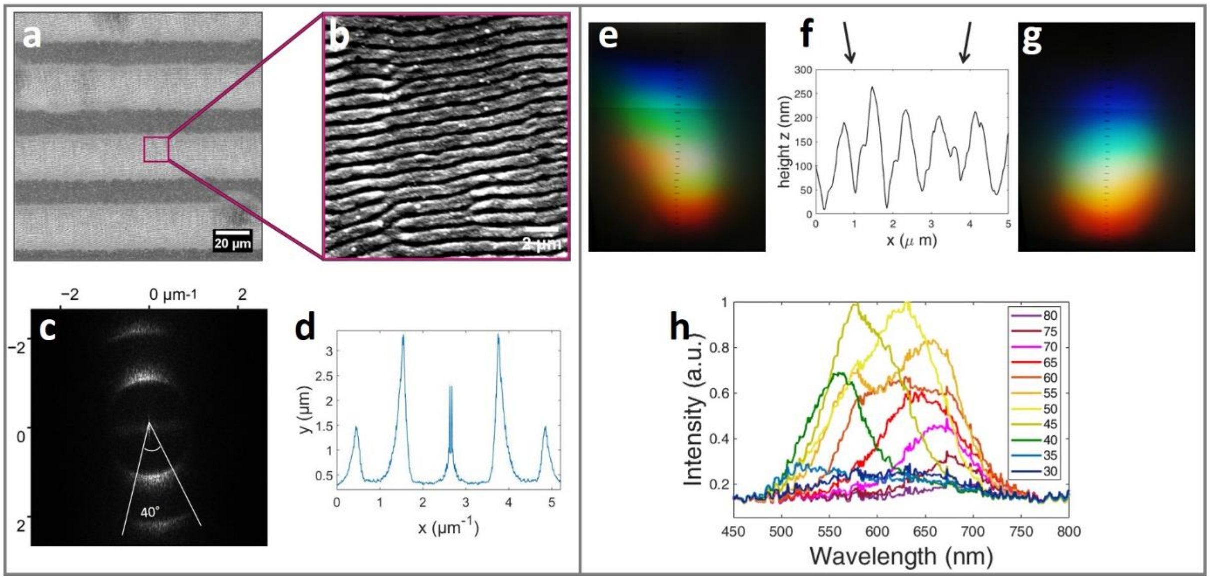

Figure S10: Area D (see table S1) 65% covered with ripples induced by N=14 femtosecond laser pulses per spot in the horizontal direction and no overlapping in the vertical direction. The LIPSS period is approx. 900 nm. Detailed description of each image is given in caption of Fig. S4.

Area **E**, 100% covered with LIPSS without vertical overlapping

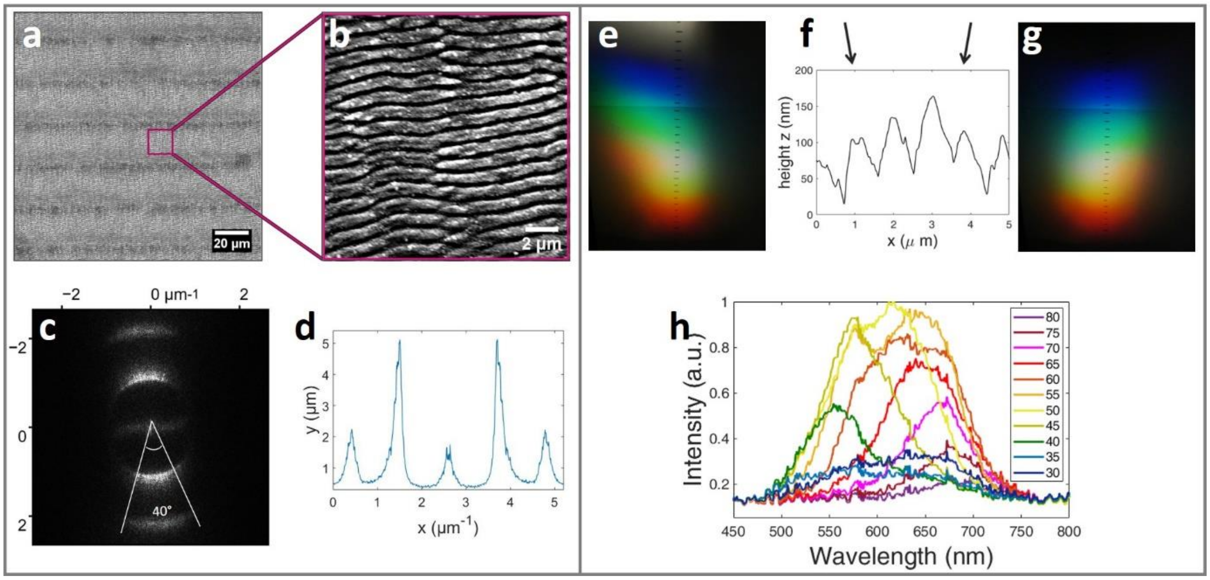

Figure S11: Area E (see table S1) 100% covered with ripples induced by N=14 femtosecond laser pulses per spot in the horizontal direction. The LIPSS period is approx. 900 nm. Detailed description of each image is given in caption of Fig. S4.

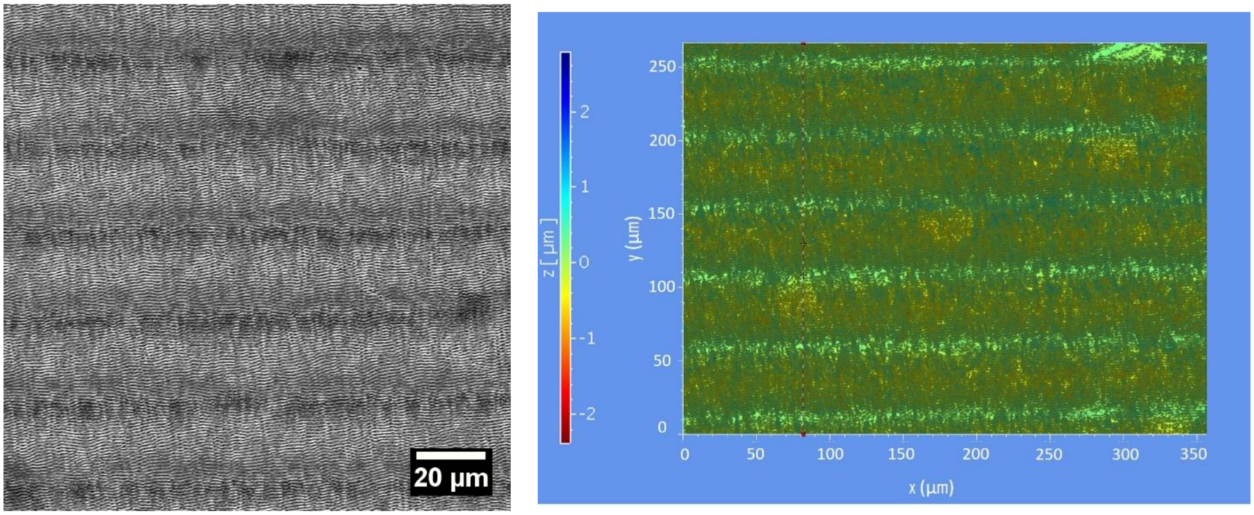

Figure S12: SEM micrograph (left) and white-light interferometry of the same area (right). (left) The same micrograph as in Fig. S11a. Each scanning line has a width of 24μm. The same is observed also for areas B, C and D. In area E the vertical step of the scanner was chosen at 24μm in order to create a surface fully covered with ripples with zero vertical overlapping. The edges of each scanning line can be observed here and also through the depth profile of the surface(right). The color map on the left represents the depth of the surface.

A closer look to the quality of the 2-D and 3-D LIPSS morphology is shown in the AFM analysis. The sinusoidal shape of their cross-section is also shown. The areas B, D and E (see Figs. S6(f), S7, S10f and S11f) are much more uniform than the areas C (Figs. S8(f) and S9) and A (Figs. S4(f) and S5), where the height of the valleys can change from period to period and the surface on top of the valleys is much rougher. Further in areas B, D and E although the ripples are finer, the orientation can slightly vary between the ripples within the same area. This disorder in the orientation and the spatial distribution of the periodic structures could be responsible for the resulting colorization. The rainbow-like colors in the photographs (Figs. (e) and (g) in S6, S10 and S11) appear more mixed which is the result of overlapping between two different visible spectra.

In addition, it is worth to notice that, in area E where 100% of the surface is covered with LIPSS, there is no vertical overlapping as in area A (Fig. S4) and as seen in the SEM pictures (see Fig.S12), one can observe the points at which two scanning lines connect in area E.

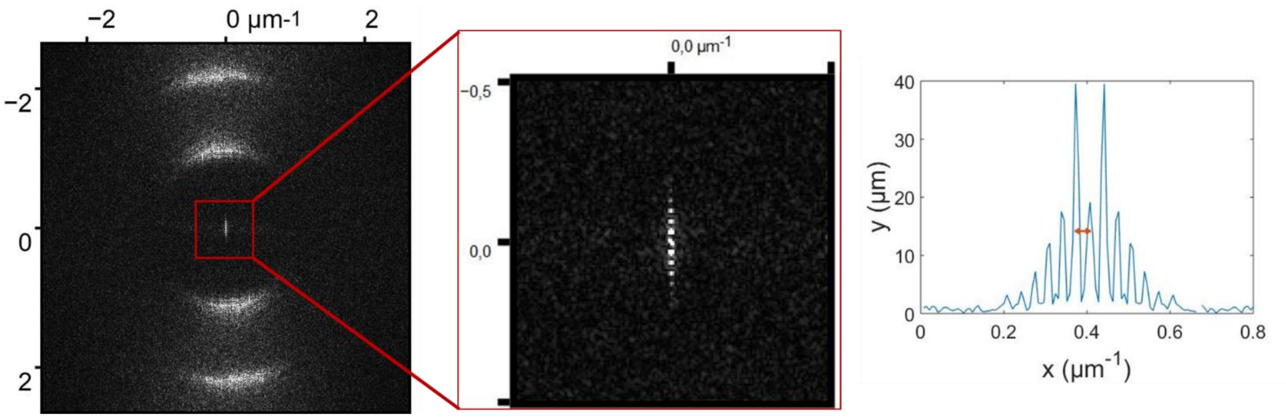

Figure S13: FFT of area B (see Fig. S6). The central area (inside red box) is magnified (middle image) showing the k vector of the low spatial frequencies. These low spatial frequencies are due to the repeated non-irradiated areas between the scanning lines. The width between the zero frequency and the first peak as illustrated with an orange arrow gives the frequency f which corresponds to 0.033μm^{-1}. This is inversely proportional to the average ripple period. Therefore, the period of the non-irradiated areas is 30.3μm, very close to the distance of lines (or width) given in Table S1 for the area B.

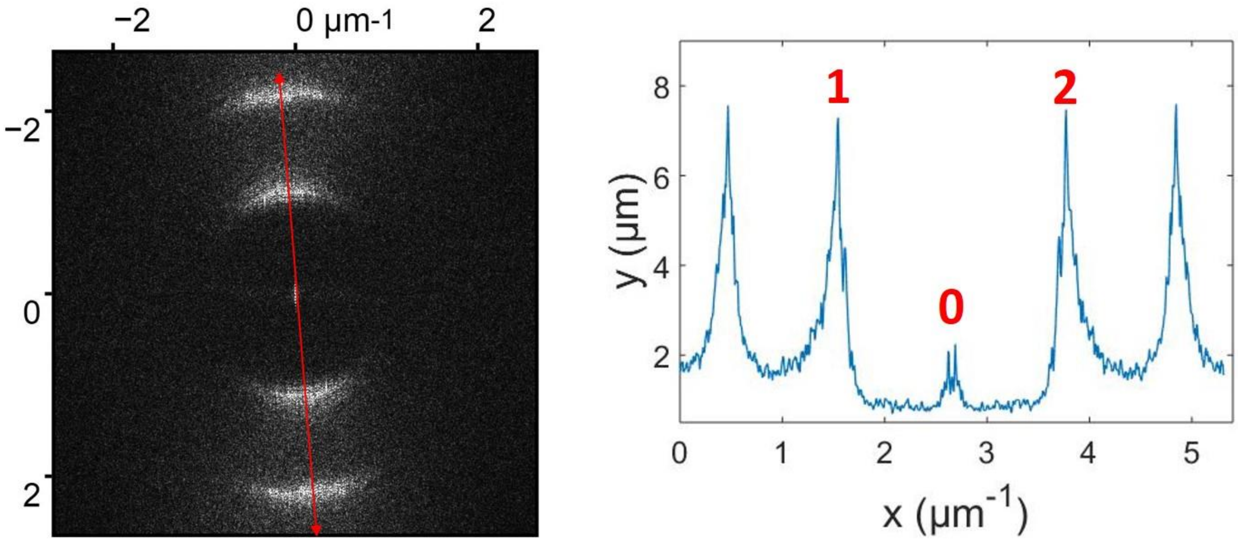

Figure S14: 2D FFT (left) of area B (see Fig. S6) and the 2D-FFT profile (right) of the red line.

Two dimensional fast Fourier transform (2D-FFT)

For the calculation of the spatial period of ripples, a 2D fast Fourier transform (2D-FFT) was employed. SEM images had been transformed in reverse space images (frequency domain) via a 2D-FFT algorithm. The new dimensions of the generated Fourier images are inversely proportional to x and y dimensions of the original SEM image. Fig.S4c represents a 2D-FFT transformation of a 2D SEM image (Fig. S4a). In order to extract spatial period information, the profile of the 2D-FFT is analyzed. The red line in Fig. S14

represents the direction perpendicular to LIPSS. Along this direction the Fourier transformation detects a periodical fluctuation of the frequency intensity, which exhibits an average frequency which is inversely proportional to the average ripple period. In particular, the distance between the center of the right plot in Fig.S14 (peak pointed with 0) and the first peak (pointed with 1) represents the characteristic frequency f of the periodic structure. The same is also shown in the FFT profile of Fig. S13. The arrow indicated the frequency f, which is the width between the zero frequency and the first peak. The spatial period Λ of the structures is defined as $1/f$. For the estimation of the range of frequencies involved into the 2D-FFT images, a Lorenzian fit is applied. The error of each measurement is calculated using the following relation: $\Delta\Lambda=|-1/f^2| * \Delta f$, were Δf is the mean of the line widths for the two Lorenzian fit curves (pointed with 1 and 2 in Fig. S14) of the 2D-FFT image profile peaks.

As shown the width of the 2D-FFT profile peak and therefore the variation of frequencies, is much wider for area A (Fig. S4d) in comparison to areas B (Fig. S6d), C (Fig. S8d), D (Fig. S10d) and E (Fig. S11d). However, their amplitude is much smaller. That means that the variation of frequencies is larger, but the contribution of these twinnings and point-like defects in the visible image (Figs. S14(e) and S14(g)) is much less pronounced in comparison to the LIPSS waving.
